# Epidemiologic characterization of 30 confirmed cases of human infection with avian influenza A(H7N9) virus in Hangzhou, China

**DOI:** 10.1186/1471-2334-14-175

**Published:** 2014-03-31

**Authors:** Hua Ding, Li Xie, Zhou Sun, Qing-Jun Kao, Ren-Jie Huang, Xu-Hui Yang, Chun-ping Huang, Yuan-Yuan Wen, Jing-Cao Pan, Xiao-Ying Pu, Tao Jin, Xiao-Hong Zhou, Lin Zheng, Jian Li, Feng-Juan Wang

**Affiliations:** 1 Hangzhou Center for Disease Control and Prevention, Hangzhou 310021, China; 2 Shangcheng District Center for Disease Control and Prevention, Hangzhou 310009, China; 3 Xiacheng District Center for Disease Control and Prevention, Hangzhou 310003, China; 4 Xihu District Center for Disease Control and Prevention, Hangzhou 300013, China; 5 Jianggan District Center for Disease Control and Prevention, Hangzhou 310004, China; 6 Xiaoshan District Center for Disease Control and Prevention, Hangzhou 311201, China

**Keywords:** Human avian influenza, H7N9 subtype, Epidemiologic characteristics, External environmental detection

## Abstract

**Background:**

We examined the clinical and epidemiological characteristics of 30 cases of human infection with avian influenza A(H7N9) virus in Hangzhou and investigated their external environments to provide evidence for contact tracing and disease prevention and control.

**Methods:**

The cases confirmed from April 1 through May 1, 2013 were studied. Field epidemiologic surveys were conducted to collect the clinical and epidemiologic data. Case-related and environmental specimens were collected for etiologic detection.

**Results:**

Thirty cases of human infection with avian influenza A(H7N9) virus were confirmed in Hangzhou from April 1 through May 1, 2013, including one pregnant woman and three deaths. The median age of the patients was 62 years (range: 38–86 years). Twenty-three of the patients were men (76.67%). The median duration between disease onset and occurrence of respiratory failure and confirmed diagnosis was 5 and 6 days, respectively. Maximum medical observation of 666 close contacts of the patients revealed no irregularity. Of 314 external environmental specimens, the overall positive detection rate of H7N9 nucleic acid was 28.98%. Eight districts of Hangzhou city had positive detections in the external environments, the highest rate being in Yuhang District (78.13%). Statistical analysis of the specimen collection locations indicates a significant difference between the case-linked locations and the non-case locations (χ^***2***^ = 16.563, *p* < 0.05) in terms of H7N9 viral nucleic acid detection rate. No epidemiologic link has been found among the 30 cases.

**Conclusions:**

Most of the infected were retired individuals aged 60 years or older. Men made the majority. The cases are sporadic at present, with no evidence of human-to-human transmission. Exposures to poultry and live poultry markets may be important sources of infection.

## Background

China reported the first events of human infection with H7N9 avian influenza A(H7N9) virus in March 2013. The virus has been proved to be a novel strain, the first of its kind ever found to infect humans, which was previously found only in wild bird populations. Avian influenza has caused frequent epidemics in humans and animals in recent years, the H5N1 avian influenza in 2005 and the novel H7N9 human influenza epidemic in 2013 in particular, which draws global attention. The avian influenza viruses isolated from domestic fowl and wild birds in many Asian, African and European countries not only cause devastating damage to the avian industries but pose a severe threat to public health of humanity [1-5]. Studies have revealed such viral strains as H7N1, H7N2, H7N3, H7N4, and H7N7 to cause outbreaks of avian influenza, which led to mass culling of over 75 million domestic birds [1]. Over the last few decades, researchers have realized that avian influenza viruses may be linked with human infections, primarily transmitted from domestic birds to humans directly [3,6-9]. Infections and deaths attributed to the H9N2 [6], H7N3 [10], H7N2 [11], H7N7 [12], and H5N1 strains [7] have been reported globally. In March 2013 cases of human infection with avian influenza A(H7N9) virus were first reported in Shanghai and Anhui province of China, followed by cases in the surrounding cities and provinces. Most of the cases were severe with high fatality. By 17:00 May 1, 2013 China had reported a total of 129 confirmed cases, including 31 deaths, 42 recoveries, and 56 patients currently treated at designated facilities. By far the cases have been sporadic except for the household clusters found in Shanghai and Beijing. Of the 46 confirmed cases in Zhejiang province, 30 were found in Hangzhou city, including one pregnant woman and three deaths. In characterizing the cases to support future prevention and control, we report the clinical and epidemiologic characteristics of the 30 Hangzhou cases and their environmental test findings.

## Methods

Field epidemiologic surveys were launched to attain the patients’ clinical and epidemiologic data, including their demographic information, disease onset, medical management, clinical presentations, dwelling environments and exposures, domestic fowl keeping, living habits and customs, past histories, possible sources of infection, transmission routes, and exposure factors. Close contacts of the patients were placed on medical observation. Informed consent to participate in the study was obtained from all the involved individuals. The current work was approved by the Hangzhou Center for Disease Control Ethics Committee (approval number: 47011685-X).

The epidemiologic surveys were carried out by epidemiologists from Hangzhou city and the local districts and counties, following the Prevention and Control Protocol for Human Infections with Avian Influenza A(H7N9) (1^st^ Edition) [13] issued by the National Health and Family Planning Commission (NHFPC). Throat swabs were collected from the patients and close contacts, which were packed and shipped, following the appropriate national bio-safety regulations, to the Influenza Monitoring Network laboratory for etiologic studies within 24 hours. All of the throat swabs were collected during standard medical care.

### Definitions

Human avian influenza A(H7N9) case: the cases were categorized as suspected cases and confirmed cases according to the Diagnosis and Treatment Protocol for Human Infections with Avian Influenza A (H7N9) (1^st^ and 2^nd^ Editions, 2013) [14,15].

Suspected case: a patient whose clinical symptoms are consistent with acute influenza (fever, cough, coryza, difficulty breathing) and laboratory test result positive for infection with an untyped influenza A virus or who has a contact history with a confirmed or suspected case.

Confirmed case: a patient whose clinical symptoms are consistent with acute influenza (fever, cough, coryza, difficulty breathing) or who has a contact history with a confirmed or suspected case and a laboratory test positive for avian influenza A (H7N9) virus; PCR, viral isolation or a four-fold or greater increase in serum antibodies specific for the H7N9 virus isolated in paired sera.

Close contact: a medical worker or a relative of patient who gave care for a suspected or confirmed patient without proper personal protections; an individual who lived or had close contact with a suspected or confirmed patient between onset and isolation of the patient; and any other individual identified by field investigator to qualify for inclusion.

Case-linked location: live poultry markets in a district, which the confirmed patients had visited over the last two weeks, were chosen for collection of case-linked specimens.

Non-case location: live poultry markets in the same district, which the confirmed patients had not visited over the last two weeks, were chosen for collection of non-case specimens.

Exposure to live poultry market: visitation to a live poultry market (either a retail or wholesale market) within two weeks prior to disease onset, especially presence within 1 m from a stall where live poultry was sold or slaughtered.

Exposure to poultry: direct or indirect contact with any poultry or poultry product within two weeks prior to disease onset, including purchasing, cleaning, cooking and eating of suchproducts [16].

Environmental specimen collection: environmental specimens such as poultry feces, anal swabs, throat swabs, drinking water, waste water, and feather smears were collected.

Specimen collection location: the specimens were mainly collected from live poultry markets, poultry keeping households of patients and their adjacent environments, poultry farms and poultry keeping households adjacent to patient households. The specimen collection locations in a live poultry market included case-linked and non-case locations. At least one nearby live poultry markets were chosen as a non-case location for each case-linked location.

Severe case: a confirmed case with pneumonia complicated by respiratory failure or failure of other organs.

### Laboratory testing

Results of routine blood tests, biochemical examinations and chest radiography were attained from the medical laboratories of the designated facilities. RNeasy Mini Kit (QIAGEN) was used to extract viral RNA for etiologic studies. Real-time retroviral-transcription polymerase chain reaction (RT-PCR) (Real-time PCR System, ABI7500) was used for viral subtyping. The primers and probes for testing H7N9 viral nucleic acid were developed by the Chinese Center for Disease Control and Prevention (China CDC) according to the specific H7N9 virus gene sequences and distributed to the Influenza Monitoring Network labs all over the country.

### Statistical analysis

SPSS11.0 was used for statistical analysis. Enumeration data were expressed in frequencies and percentage. Rate differences were examined by *Pearson* χ^*2*^ test with a test level α = 0.05. The Geographic Information System (GIS) was employed to portray the spatial distribution of cases in Hangzhou.

## Results

### Epidemiologic characteristics

#### Temporal, spatial and demographic distribution of disease

**Figure 1:**
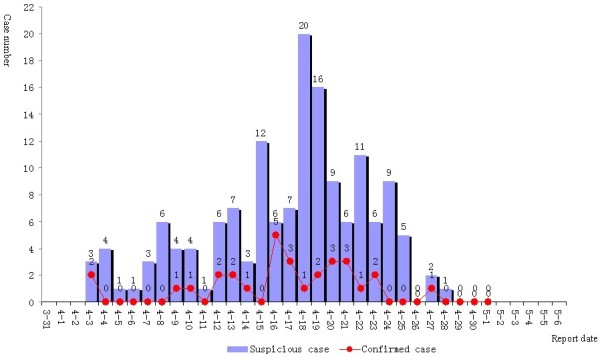
Reporting times of cases of human infection with avian influenza A(H7N9) virus in Hangzhou.

Over the 31 days by 16:00 May 1, 2013, a total of 153 cases of severe pneumonia had been reported in Hangzhou. After the cases of infection with bacteria, fungus, mycoplasma pneumoniae, chlamydia, common influenza virus, parainfluenza virus, syncytial virus or adenovirus were ruled out, the clinically suspicious H7N9 cases were subtyped with RT-PCR; 123 cases were excluded, and 30 cases of human infection with avian influenza A(H7N9) virus were confirmed. The case patients were in severe conditions with rapid progression. Three of the patients died (fatality rate: 10.00%), five were discharged on recovery, and the remaining patients were treated at designated facilities. See Figure 1.

**Figure 2:**
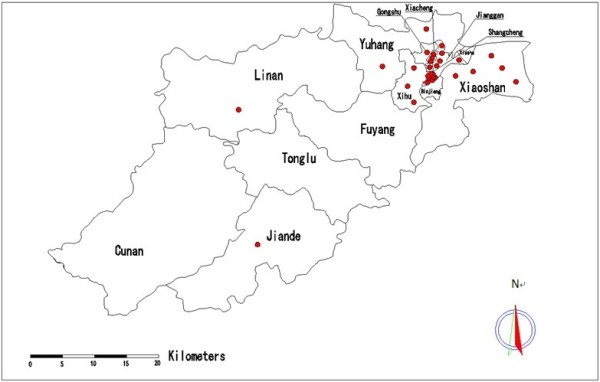
Geographic distribution of cases of human infection with avian influenza A(H7N9) virus in Hangzhou.

By May 1, 2013, H7N9 cases had been confirmed in nine of the 13 local districts and counties of Hangzhou city, including eight cases from Shangcheng District, five from Xiaoshan District, five from Jianggan District, four from Xiacheng District, three from Xihu District, two from Yuhang District, and one case from Gongshu District, Jiande County and Lin’an County, respectively. See Table 1 and Figure 2.

**Table 1:** Epidemiologic characteristics of 30 confirmed H7N9 cases in Hangzhou

**Characteristics**	**Confirmed cases (N = 30)**
Age range (median) (yr)	35.0 - 86.0 (62.0)
Male/N (%)	23 (76.67)
Occupation/N (%)	
Retired	11 (36.67)
Farmer	6 (20.00)
Industrial worker	4 (13.33)
Unemployed	4 (13.33)
Others	5 (16.67)
Residency/N (%)	
Urban	28 (93.33)
Rural	2 (6.67)
History of exposure to poultry or live poultry market/N (%)	27 (90.00)
Exposure mode/N (%)	
Both exposure to poultry and live poultry market	14 (46.67)
Only exposure to live poultry market	11 (36.66)
Only exposure to poultry	2 (6.67)
Unclear	3 (10.00)
Travel history/N (%)	3 (10.00)
Close contract/N (%)	
Medical worker	386 (57.96)
Family and relative	163 (24.47)
Others	117 (17.57)

**Figure 3:**
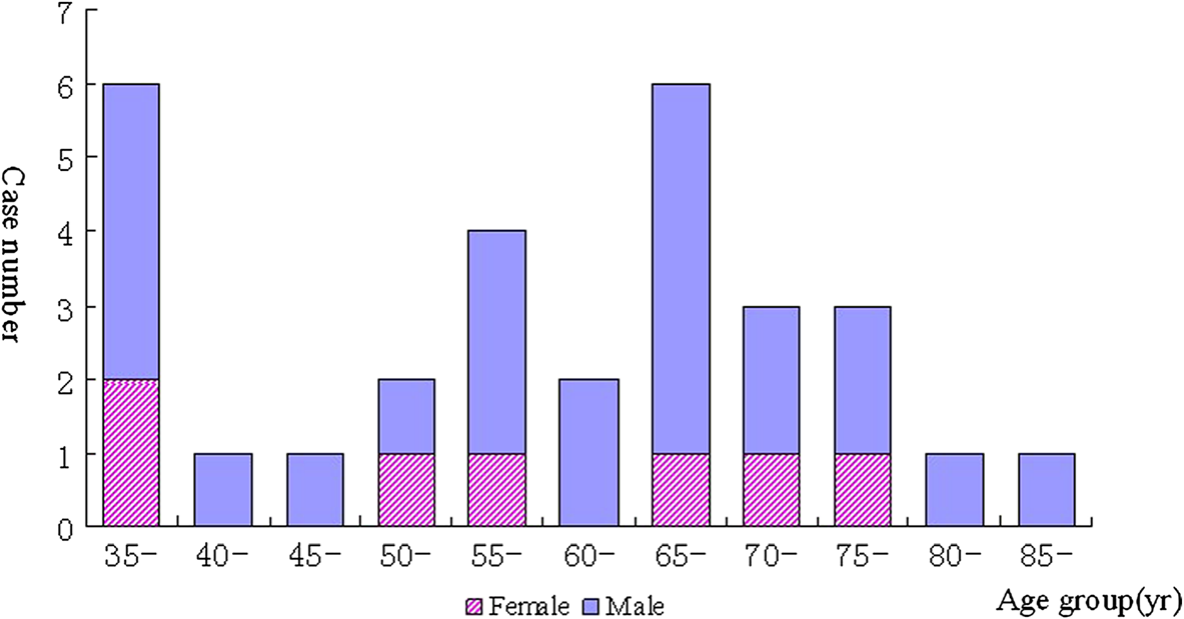
Gender and age distributions of cases of human infection with avian influenza A(H7N9) virus in Hangzhou.

Twenty-three of the 30 confirmed case patients were men (male:female ratio: 3.29:1). The patients’ ages ranged from 35–86 years (median: 62.0 years). A large fraction of the patients were retired (11 cases, 36.67%), followed by farmers (6 cases, 20.00%) by occupation. See Table 1 and Figure 3.

#### Exposure history

Twenty-seven (90.00%) of the 30 confirmed patients had definitive exposure histories, including 14 patients with exposures both to poultry and to live poultry markets, 11 only to live poultry markets, and two only to poultry. The remaining three patients’ exposure histories were indefinitive (One of the patients denied any exposure in his residential environment though the ground floor of the building he lived in was a live poultry market. The other two had no definitive histories of exposure to poultry or live poultry market). Analysis of travel histories revealed that three patients had traveled to other places, including two going to the neighboring Jiangsu province and one to a neighboring city in Zhejiang province where he had contact with poultry locally. See Table 1.

#### Close contacts

A total of 666 close contacts were identified, including 377 medical workers, 160 family members and relatives and 129 other individuals. During a 7-day medical observation and health follow-up, three of the close contacts had a fever and 47 exhibited respiratory symptoms such as sore throat and cough. Test results of the 50 symptomatic close contacts were negative for H7N9 avian influenza A(H7N9) viral infection. During the medical observation 164 throat swab specimens were collected from the asymptomatic close contacts, and the test results were negative for H7N9 viral nucleic acid. The medical observation was lifted after seven days.

### Clinical characteristics

#### Clinical characteristics, diagnosis and management

The initial symptoms of the 30 confirmed cases were primarily cough and fever (25/30 cases, 83.33%). The median time was 1 day between onset and presentation to a clinic, 4 days between onset and first hospitalization, 5 days between onset and occurrence of respiratory failure, and 6 days between onset and confirmed diagnosis. Diagnosis of the first case was confirmed five days following the patient’s death. See Table 2.

**Table 2:** Clinical features, diagnosis and management of 30 confirmed H7N9 cases in Hangzhou

**Variable**	**Confirmed case (N = 30)**
Underlying disease/(N,%)	21 (70.00)
Symptom severity/(N,%)	
Mild	14 (46.67)
Moderate	12 (40.00)
Severe	4 (13.33)
Use of artificial liver/(N,%)	7 (23.33)
Use of artificial lung/(N,%)	5 (16.67)
Use of dialysis/(N,%)	9 (30.00)
Outcome/(N,%)	
Discharged	5 (16.67)
Improved	3 (10.00)
Stabilized	17 (56.67)
Aggravated	1 (3.33)
Died	3 (10.00)
Unknown	1 (3.33)
Duration between disease onset and	
First hospital visit/median (range, days)	1 (0–19)
First hospitalization/median (range, days)	4 (0–19)
Transfer to provincial-level hospital/median (range, days)	5 (3–21)
Occurrence of respiratory failure/median (range, days)	5 (3–11)
Confirmed diagnosis/median (range, days)	6 (4–21)

#### Incubation period

Twelve of the 30 confirmed cases had definitive times of exposure to live poultry and/or live poultry market, nine of whom had single exposure time points (incubation period: 1–14 days, median: 7 days) and three had continual exposure time points before onset (incubation period: 1–7 days in two cases, 4–7 days in one case). Of the remaining 18 cases, 15 had continual exposures between onset and hospital admission, whose exposure times could not be established for incubation period calculation. No definitive history of exposure to live poultry market was documented for the other three cases.

### External environmental detection

#### Overall viral detection in live poultry and environmental specimens

A total of 314 specimens were collected from the environments of live poultry markets, large poultry farms and poultry keeping households in Hangzhou, including 159 specimens (50.64%) from the live poultry markets, 83 (26.43%) from the poultry keeping patietn households, 48 (15.29%) from the poultry farms and 24 (7.64%) from other sources. Ninety-one specimens (28.98%) tested positive for H7N9 viral nucleic acid, most of which were from the live poultry markets.

#### Viral detection in different types of specimens from live poultry markets

See Table 3 for positive detections in different types of specimens from the live poultry markets. Of the 159 live poultry market specimens, 80 tested positive (overall positive rate: 50.31%) for H7N9 avian influenza viral nucleic acid. According to Table 3, among the specimens collected from the markets, the case-related ones saw a markedly higher positive rate compared with the non-case specimens, where the *Pearson* χ^***2***^ test results indicate a statistically significant difference (χ^***2***^ = 16.563, *p* < 0.05). Another 17 throat swab specimens were collected from exposed poultry workers, all of which tested negative for H7N9 viral nucleic acid. None of the 17 poultry workers exhibited flu-like respiratory symptoms such as fever and cough.

**Table 3:** Detection of H7N9 nucleic acid in environmental specimens from live poultry markets

**Type of specimen**	**Case-related specimens**			**Non-case specimens**		
	**Tested N**	**H7N9+ N**	**+ rate (%)**	**Tested N**	**H7N9+ N**	**+ rate (%)**
Feces	13	10	76.92	10	2	20.00
Anal swab	15	9	60.00	-	-	-
Throat swab	10	5	50.00	1	1	100.00
Drinking water	-	-	-	8	3	37.50
Waste water	13	5	38.46	6	2	33.33
Feather smear	16	11	68.75	5	2	40.00
Cage smear	7	5	71.43	17	5	29.41
Others	16	13	81.82	22	7	31.82
Total	90	58	64.44	69	22	31.88

Note: −: no specimen of the type available; in the specimens collected from the live poultry markets, the positive rate for H7N9 nucleic acid was markedly higher in the case-related specimens than in the non-case ones with a statistically significant difference by *Pearson* χ^***2***^ test (χ^***2***^ = 16.563, *p* < 0.05).

#### Environmental viral detection in local districts and counties

So far H7N9 avian influenza nucleic acid has been detected in the environments in eight local districts and counties, with the highest positive detection rate in Yuhang District (78.13%).

#### Disease onset after suspension of live poultry trade

Live poultry trade was suspended throughout Hangzhou city on April 16, with the environments in the markets disinfected for seven consecutive days. The last case was reported on April 27. The exposure times of the previous cases were reportedly prior to April 16, suggesting the control measures to be effective.

### Influenza surveillance

**Figure 4:**
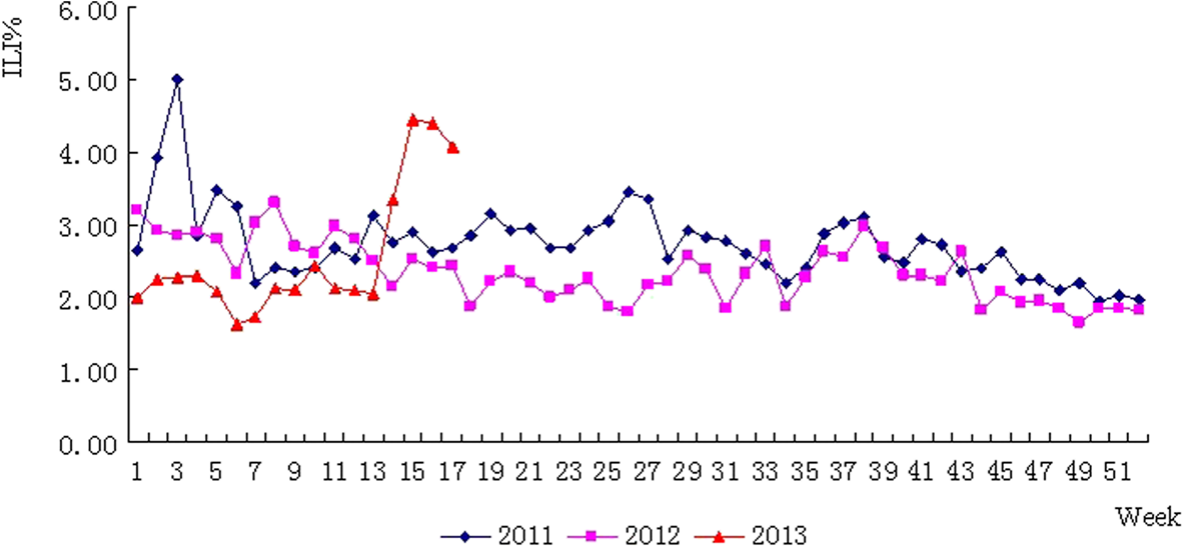
Percentage of cases of influenza-like illness: Hangzhou, 2011–2013.

There are 14 influenza surveillance sentinels in Hangzhou, including two national surveillance hospitals and 12 designated hospitals at the city level. The flu surveillance findings over Weeks 14–17 (from March 31 through April 27) indicate that the percentage of influenza-like illnesses (ILI%) rose considerably on Weeks 14 and 15 and dropped slightly on Weeks 16 and 17, without an outbreak of cluster cases. A marked increase is noted when compared with the same period two years ago. A total of 110 specimens from mild flu-like cases were sent to the sentinel facilities for testing on Weeks 14–17, none of which tested positive for H7N9 influenza, 7.27% were found with H1N1 and 8.18% with H3N2 avian influenza viruses. See Figure 4.

## Discussion

A total of 30 confirmed cases of human inflection with avian influenza A(H7N9) virus were reported in Hangzhou between April 1 and May 1, 2013, including 12 moderate and four severe cases. The clinical characteristics were consistent with the other cases reported in China [17-19]. The time series of disease progression indicate rapid progressive aggravation of severe pneumonia. The results of the current study are in alignment with the median duration before hospitalization reported by QIAN Fang, where the patients also experienced rapid progression [20].

As a newly emerging infectious disease, human infection with H7N9 avian influenza virus has drawn high attention in the professional and international communities. Timely detection is of grave importance for prevention and control measures. The 30 cases of human inflection with avian influenza A(H7N9) virus in Hangzhou were found by medical facilities through surveillance and screening of pneumonia of unknown cause and reported via the Online Direct Reporting System.

In further understanding the possible sources of infection and assessing risks of human infection, environmental specimens were collected from the live poultry markets that the patients and their family had visited as well as from poultry workers, close contacts of patients, and flu-like cases for etiologic studies. *Pearson* χ^***2***^ test results indicate a statistically significant difference (χ^***2***^ = 16.563, *p* < 0.05) between the case-related environmental specimens and the non-case specimens in terms of H7N9 viral nucleic acid positive rate. None of the close contacts manifested progressively aggravating flu-like or pneumonic symptoms. This suggests that virus activity may exist in absence of an animal outbreak in the live poultry markets. Information on the virus-bearing animals, contaminated environments, pathogenic homology and the exposure history of population makes possible a general portrait of the chain of transmission. Testing and medical observation of close contacts have so far yielded no evidence of human-to-human transmission. Etiologic monitoring of outpatients with flu-like symptoms has documented no mild cases of H7N9 infection. However, as the monitoring employed throat swabbing, the results might be less sensitive compared with serologic testing and further evidence, serologic test results for example, may be needed.

Exposure mode investigation indicates that most patients were retired elderlies, who visited live poultry markets on a daily basis to purchase food ingredients, including live poultry products. It is a common practice in China that live poultry is slaughtered on site of purchase in the market to ensure freshness, which is believed to keep the food tasty. As a result, it was likely that the patients were repeatedly exposed in the less hygienic markets for significant periods of time, who were in immediate contact with live fowl and freshly slaughtered poultry products without appropriate nose and hand protections. Sustained exposures in the live poultry markets makes calculation of incubation period difficult. Fifteen of the Hangzhou cases had continual exposures between disease onset and hospitalization, where the exact valid exposure time points could not be determined to calculate the incubation periods. However, definitive time points of exposure to live poultry and/or the environments of live poultry markets before disease onset were available for 12 cases, including nine cases with single exposure time points, based on which the incubation period was calculated to be 1–14 days (median: 7 days), which is longer than that of avian influenza H5N1 in China. A study by HUAI Y indicates that based on the exposure and onset data of 24 confirmed H5N1 cases, the median incubation period of H5N1 avian influenza in China is calculated to be 5 days (range: 2–9.5 days) [21]. A more accurate incubation period of H7N9 may be determined by investigating the 127 confirmed cases countrywide in China.

At the moment, H7N9 cases are sporadic and scattered events except for the household clusters found in Shanghai and Beijing, and the main source of infection remains unclear [22]. The current work may not have covered all possible sources of infection. Despite a thorough infection source investigation carried out by a joint survey team, the sources of three confirmed cases remain undetermined. A similar situation was seen in an H5N1 study in Guangdong province, China [23].

The H7N9 PCR positive rate was the highest in the environmental specimens from Yuhang District, where only two cases were confirmed. In contrast, the other districts saw more cases despite lower environmental detection rates, Shangcheng District for instance, where eight cases were found while the environmental positive rate was lower. Yuhang is the largest district of Hangzhou city, where two major designated markets supply more than half of the live poultry for the rest of the city. Compared with Yuhang District, the population in the other districts is less dense. Additionally, the live poultry vendors are mixed with other food stalls, which the people in the other district visit on a daily basis. This might explain the higher environmental detection rate with fewer cases in Yuhang District.

According to the existing data, the main route of transmission is believed to be avian-to-human. The sources of infection could be exposures to live poultry markets. The mode of infection remains unclear. Humans generally lack immunity to the novel infectious disease and effective immunization is currently unavailable. Therefore, cutting of the chain of transmission and effective environment disinfection and isolation measures seem particularly important.

### Limitations

There are two limitations to the current work: 1. environmental specimen collection in the districts where cases were found earlier might have been affected by control interventions. For instance, early regular disinfection of live poultry markets and other control measures took place in Shangcheng District. This might have caused sampling errors; and 2. the study was not case–control, which may be conducted in future outbreaks to determine the risk factors.

## Conclusions

Most of the infected were retired individuals aged 60 years or older. Men made the majority. The cases are sporadic at present, with no evidence of human-to-human transmission. Exposures to poultry and live poultry markets may be important sources of infection.

## Competing interests

The authors declare no conflict of interest in the current work.

## Authors’ contribution

HD led the survey of and response to the H7N9 outbreak in Hangzhou and coordinated the district and county CDC and hospitals for on-site epidemiological survey data and specimen collection. LX is the director of the Agency for Communicable Disease Prevention and Control, Hangzhou CDC, who led and coordinated the survey of and response to the H7N9 outbreak in Hangzhou and organized data input and manuscript writing. ZS participated in the on-site epidemiological survey and specimen collection. QJK participated in the on-site epidemiological survey and specimen collection. RJH participated in the on-site epidemiological survey and specimen collection. XHY participated in the on-site epidemiological survey and specimen collection. CPH aggregated and reported the outbreak information. YYW participated in the on-site epidemiological survey and specimen collection, input the data and drafted the manuscript. JCP is the director of the Microbiology Laboratory, Hangzhou CDC, who performed the tests including the sequence alignment. XYP performed the tests including the sequence alignment. TJ participated in the on-site epidemiological survey and specimen collection. XHZ participated in the on-site epidemiological survey and specimen collection. LZ participated in the on-site epidemiological survey and specimen collection. JL participated in the on-site epidemiological survey and specimen collection. FJW participated in the on-site epidemiological survey and specimen collection. All authors read and approved the final manuscript.

## Pre-publication history

The pre-publication history for this paper can be accessed here:

http://www.biomedcentral.com/1471-2334/14/175/prepub
